# Triptolide-mediated downregulation of FLIP_S_ in hepatoma cells occurs at the post-transcriptional level independently of proteasome-mediated pathways

**DOI:** 10.1007/s12032-022-01857-y

**Published:** 2022-10-29

**Authors:** Weixia Liu, Ying Yang, Jing Wang, Shanshan Wu, Zhi Chen

**Affiliations:** State Key Laboratory for Diagnosis and Treatment of Infectious Diseases, National Clinical Research Center for Infectious Diseases, National Medical Center for Infectious Diseases, Collaborative Innovation Center for Diagnosis and Treatment of Infectious Diseases, The First Affiliated Hospital, Zhejiang University School of Medicine, Hangzhou, China

**Keywords:** Triptolide, FLIP_S_, Death receptor ligand, Apoptosis, ROS, Proteasome

## Abstract

Cellular c-FLIP prevents apoptosis mediated by death receptor through inhibiting activation of caspase-8. Therefore, when c-FLIP is downregulated or eliminated, caspase-8 activation is promoted, and death receptor ligand-induced apoptosis is activated. It was reported that triptolide (TPL) sensitized tumor cells to TNF-α-induced apoptosis by blocking TNF-α-induced activation of NF-κB and transcription of c-IAP1 and c-IAP2. However, the effect of TPL on basal c-FLIP expression was not understood. In this study, we found that the combination of TNF-α and TPL accelerated apoptosis in human hepatocellular carcinoma cells and TNF-α-induced elevated as well as basal level of FLIP_S_ protein were downregulated by TPL. Additionally, we demonstrated that the basal level of FLIP_S_ in Huh7 cells was continuously downregulated following the incubation of TPL and downregulated more when dosage of TPL for treatment was increased. Subsequently, we showed that TPL reduced FLIP_S_ level in a transcription- and degradation-independent mechanism. Our findings suggest that TPL induces loss of FLIP_S_ at the post-transcriptional level independently of proteasome-mediated pathway, an additional mechanism of TPL sensitizing cancer cells to TNF-α-induced apoptosis.

**Supplementary Information:**

The online version contains supplementary material available at 10.1007/s12032-022-01857-y.

## Introduction

Previous study demonstrated that triptolide (TPL) promoted TNF-α-induced apoptosis in solid tumor cells such as A549 (nonsmall cell lung cancer), HT1080 (fibrosarcoma), and MCF-7 (breast cancer) by inhibiting activation of NF- κB and blocking transcription of c-IAP1 and c-IAP2 mRNA [1]. However, apart from these mechanisms, there was no conclusion on other mechanisms of TPL sensitizing the solid tumor cells to TNF-α-induced apoptosis.

Cellular Fas-associated death domain-like interleukin 1β-converting enzyme inhibitory protein (c-FLIP) is upregulated by activated NF-κB in diverse cancer cells, helping for apoptosis resistance [2, 3]. FLIP_L_, FLIP_S_, and FLIP_R_ are three isoforms of c-FLIP [4–6]. Like FLIP_L_, FLIP_S_ has two tandem amino (*N*)-terminal death-effector domains. Since c-FLIP binds to FADD via the death-effector domains, preventing precursor of caspase-8 from binding to FADD and causing death receptor-mediated apoptotic cascade to break [4], several chemical compounds could make cancer cell lines sensitive to tumor necrosis factor-related apoptosis-inducing ligand (TRAIL)-induced apoptosis by targeting c-FLIP protein for proteasome-dependent degradation [7–13]. Unlike these chemicals, in prostate cancer cells, doxorubicin induced the downregulation of FLIP_S_ by a post-transcriptional mechanism which did not involve proteasome [14, 15]. Previously, a study revealed that TPL reduced c-FLIP protein levels in pancreatic cancer cells and sensitized the pancreatic cancer cells to TRAIL-induced activation of apoptosis [16]. But the mechanism by which TPL reduced c-FLIP protein levels was not revealed.

Like other solid tumor cells, most human liver tumor cells possess mutations in p53 gene, which resist chemotherapy. We show here that TNF-α combined with TPL induces apoptosis in human hepatocellular carcinoma cells with mutant p53. And TPL downregulates not only TNF-α-induced elevated but also basal level of FLIP_S_. Besides, our results indicate that TPL appears to downregulate basal FLIP_S_ expression by a proteasome-independent mechanism at the post-transcriptional level, which may be correlated with the effects of TPL on tumor cells.

## Materials and methods

### Compound

TPL (molecular formula, C20H24O6) was purchased from Shanghai Tongtian Biotechnology Co., Ltd. The material was composed of white powder and 97% pure by HPLC determination.

### Cell culture

Human hepatocellular carcinoma cell lines Huh7 and Hep3B were grown in Dulbecco’s modified Eagle's medium (HyClone) supplemented with 10% fetal bovine serum, 100 units/ml penicillin, and 100 ug/ml streptomycin.

### Analysis of Huh7 cell death induced by TPL and/or TNF-α

Recombinant human TNF-α was purchased from Peprotech. Death of Huh7 cells induced by TPL and/or TNF-α was analyzed by Cell counting kit 8 (CCK-8) (Dongren Chemical Technology Co., Ltd., Shanghai). Briefly, cells were seeded in a 96-well plate (3 × 10^3^ cells/well) and then treated with different concentrations of TPL or TNF-α (0, 2.5, 5, 10, 20 ng/ml) or a combination of TPL (5 ng/ml) and the precedent concentrations of TNF-α for 48 h. Afterward, all the treated cells were incubated with CCK-8 solution in the cell incubator for another 3 h. Then, a microplate reader (Bio-Rad, Hercules, CA) was used to measure the absorbance at 450 nm.

Besides, Huh7 cells were seeded in a 6-well plate at a density of 1.7 × 10^5^ cells per well and cultured overnight. After the cells were attached, TPL and/or TNF-α was added in the culture medium to incubate cells for approximately 43 h, a time point at which death of the cells was visible. Thereafter, the cells were harvested for determination of the protein c-FLIP as well as proteins participating in apoptosis.

### Treatments of cells for analysis of FLIP_S_ downregulation promoted by TPL

Huh7 cells were maintained in mediums containing 0, 5, 10, 20, 25 ng/ml TPL for 24 h or 48 h before being harvested for determination of c-FLIP protein and mRNA. To confirm the effects of TPL on c-FLIP protein expression were not cell line specific, Hep3B cells were also incubated with TPL for 24 h followed by analysis of c-FLIP levels by Western blot. Besides, Huh7 cells were incubated with medium containing 20 ng/ml TPL for 0, 6, 12, 22, 32 h and then collected for evaluation of c-FLIP protein levels.

Moreover, Huh7 cells were untreated or pre-treated with proteasome inhibitor Lactacystin (LC) (APExBIO Technology LLC, Houston) or MG132 (MedChemExpress, Shanghai) for 2 h before the addition of TPL, and 4 h, 8 h, 12 h (for cells pre-treated with LC) or 2 h, 4 h, 6 h (for cells pre-treated with MG132) after TPL was added in, cells were harvested for analysis of FLIP_S_ levels.

### Real-time PCR

Total RNA of the cell was extracted and purified with RNAiso Plus (TaKaRa, Otsu, Japan) and chloroform. Then cDNA was obtained from reverse transcription reaction using PrimeScript RT reagent Kit with gDNA Eraser (TaKaRa). Real-time PCR were carried out on ABI PRISM 7900HT/FAST (Applied Biosystems, Foster, CA) at 40 cycles of 95 ℃ for 5 s and 60 ℃ for 30 s. Primers for FLIP_L_ were 5′-GGCTCCCCCTGCATCAC-3′ and 5′-TTTGGCTTCCCTGCTAGATAAGG-3′. Primers for FLIP_S_ were 5′-ACCCTCACCTTGTTTCGGACTAT-3′ and 5′-TGAGGACACATCAGATTTATCCAAA-3′. Levels of both isoforms were normalized to GAPDH and fold change in the level of each isoform between treated and control group was calculated with the 2^−ΔΔCt^ method. GAPDH primers were: 5′-GGAGCGAGATCCCTCCAAAAT-3′ and 5′-GGCTGTTGTCATACTTCTCATGG-3′.

### Western blotting

The harvested cells were lysed in RIPA buffer (50 mM Tris-Cl, pH 8.0, 150 mM NaCl, 1 mM EDTA, 1 mM EGTA, 0.1% SDS, 0.5% deoxycholic acid, 1% Triton X-100) supplemented with 10% PMSF. Protein concentration of cell lysate was determined using BCA protein assay kit (Pierce). Equivalent amount of protein (50 ug) was fractionated by precast mini polyacrylamide gels (SurePAGE™, Bis-Tris, 4–20%) (GenScript, Nanjing, Jiangsu) and undergone western blotting. The proteins were visualized by enhanced chemiluminescence (Proteintech Group, Inc., Chicago, IL) according to the manufacturer’s instructions.

### Determination of ROS level

The cellular ROS was detected by fluorescent probe DCFH–DA (Beyotime, Shanghai, China). Briefly, cells were inoculated into 6 -cm dishes at a density of 1 × 10^6^ cells/dish, incubated overnight, and then treated with 20 ng/ml of TPL for 24 h. After that, the culture medium was changed into 2 ml DMEM containing 10 uM of DCFH–DA and the cells were incubated for another 1 h in the incubator. Then the cells were washed 3 times with DMEM to fully remove the left DCFH–DA which did not enter the cell. Finally, fluorescence intensity in each cell was determined by the flow cytometer on which 488 nm was used as the excitation wavelength and 525 nm was used as the emission wavelength. Mean fluorescence intensity (MFI) representing the level of ROS was quantitated with Flowjo10 software.

## Results

### TPL made Huh7 cells sensitive to TNF-α-induced apoptosis

As shown in Supplemental Table 1, after 48 h of treatment with TNF-α or/and TPL, Huh7 cells were resistant to TNF-α and approximately 90% of Huh7 cells remained viable against 5 ng/ml TPL, but less than 70% of Huh7 cells survived from the combination of TNF-α and TPL (5 ng/ml).

Apoptosis induced by the combination of TNF-α and TPL in Huh7 cells was represented by the appearance of active caspase-8, caspase-3, and cleaved PARP (Supplemental Fig. 1).

### TPL treatment reduced basal and TNF-α-induced elevated level of FLIP_S_

**Fig. 1:**
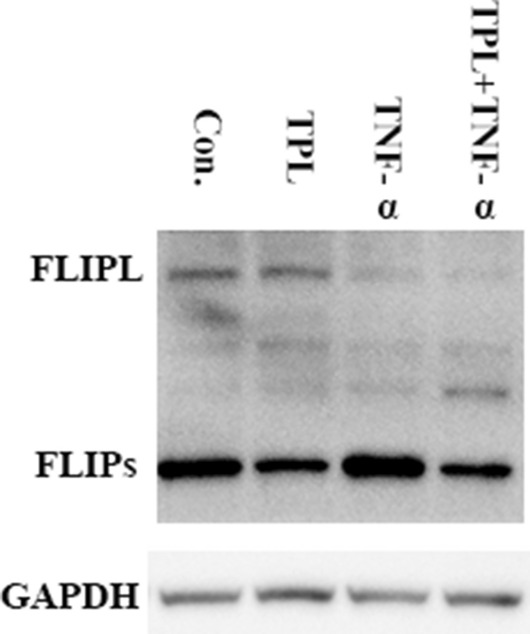
Treatment of Huh7 cells with TPL or the combination of TNF-α and TPL brought about downregulation of FLIP_S_ protein levels. Huh7 cells were treated with 5 ng/ml TNF-α or/and 5 ng/ml TPL for approximately 43 h. Total cell lysates of the treated cells were analyzed with a specific rabbit anti-FLIP antibody via Western blot. Identical anti-FLIP antibodies were used in the following Western blot experiments unless otherwise stated

In view that downregulation of cellular endogenous c-FLIP protein levels sensitized tumor cells to death receptor-mediated apoptosis, we determined c-FLIP protein levels in Huh7 cells untreated or treated with TNF-α and/or TPL. Our result showed that FLIP_S_ was the predominant isoform of c-FLIP in Huh7 cells and FLIP_S_ levels were enhanced by TNF-α but reduced by TPL treatment (Fig. 1). In addition, FLIP_L_ levels were reduced by TNF-α, and in Huh7 cells treated with TNF-α and TPL, not only the increase in FLIP_S_ induced by TNF-α was blocked but also basal level of FLIP_S_ was downregulated.

### FLIP_S_ levels were continuously downregulated following the increasing dosage and time of TPL for treatment

**Fig. 2:**
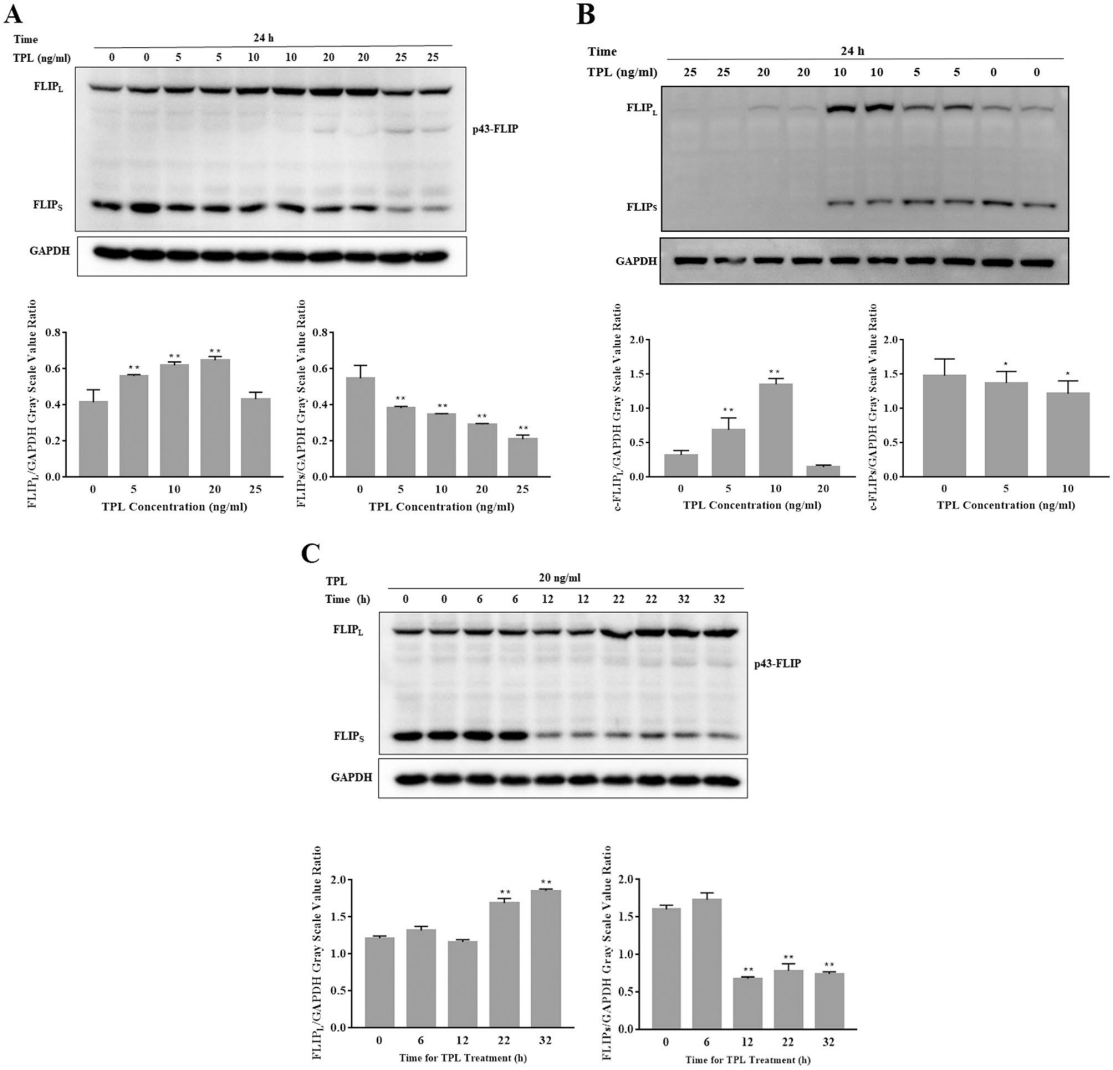
c-FLIP protein expression in hepatocellular carcinoma cells treated with TPL. FLIP_L_ and FLIP_S_ proteins were determined by Western blot from Huh7 (**A**) or Hep 3B (**B**) cells treated with TPL at various concentrations for 24 h or Huh7 cells treated with 20 ng/ml TPL for the times indicated (**C**). Expression of c-FLIP was quantitated by densitometry and normalized against that of GAPDH, respectively. The relative expression of c-FLIP was presented as mean ± S.D. calculated from three independent experiments; *P < 0.05 or **P < 0.01, compared with 0 ng/ml (in **A** or** B**) or 0 h (in **C**)

Subsequently, we further investigated the effects of TPL on inducing decrease in FLIP_S_ levels in Huh7 and Hep3B cells. Both cell lines were treated with TPL at various concentrations for 24 h. Then the expression of c-FLIP protein was determined with Western blot (Fig. 2A, B). Treatment with TPL (5/10 ng/ml) increased the expression of FLIP_L_ protein in a dose-dependent manner. In contrast, the protein level of FLIP_S_ was reduced after 24 h of TPL treatment at concentrations ranging from 5 to 25 ng/ml. Additionally, time-course experiments were performed in incubating Huh7 cells with TPL to investigate the effects of TPL on the expression of c-FLIP protein. Figure 2C shows that FLIP_L_ levels were significantly enhanced after 22 or 32 h of treatment with TPL even though a part of the FLIP_L_ proteins were cleaved to produce a protein, p43-FLIP. Conversely, FLIP_S_ levels were reduced, as a matter of fact, as early as 12 h of TPL treatment, the level of FLIP_S_ protein was lowered strikingly. Our results confirm that the FLIP_S_ protein expression is inhibited by treatment with TPL.

### TPL increased the c-FLIP mRNA level

**Fig. 3:**
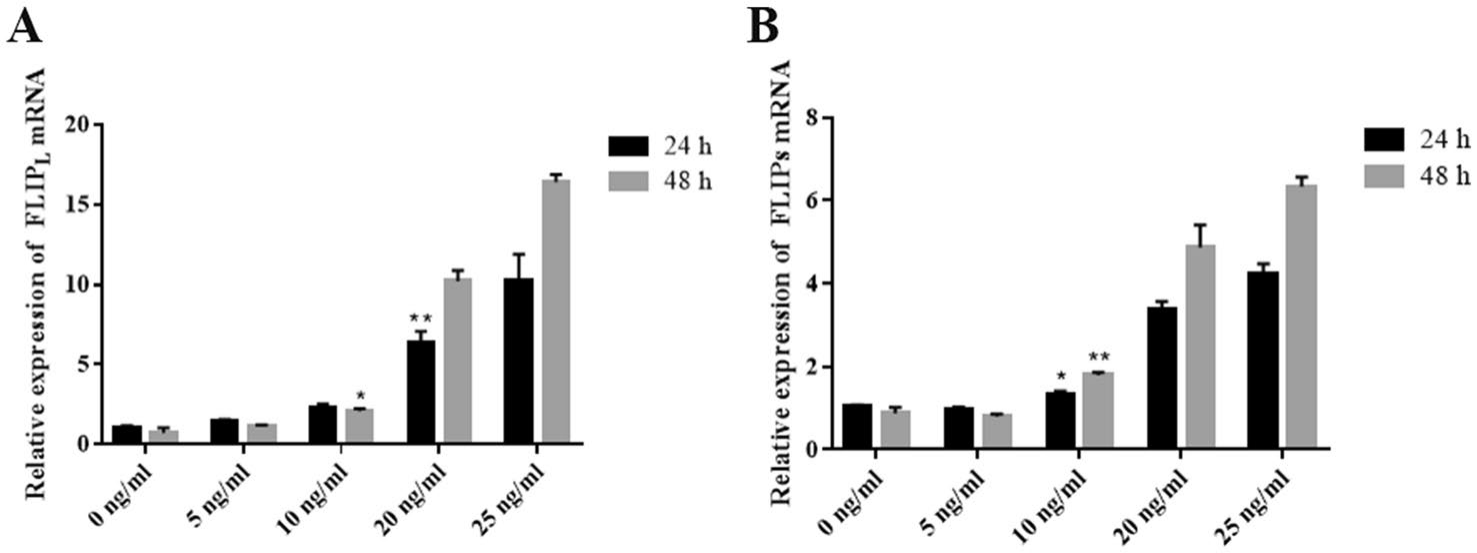
Expression of c-FLIP mRNA in Huh7 cells treated with TPL. TPL at concentrations ranging from 5 to 25 ng/ml was used for treating Huh7 cells for 24 h or 48 h. The mRNA level of c-FLIP was analyzed by relative quantitative RT-PCR and GAPDH was used as an internal reference. Relative expression of c-FLIP mRNA was expressed as mean ± S.D. from three independent experiments; *P < 0.05, **P < 0.01, compared with 0 ng/ml. **A** FLIP_L_ mRNA expression; **B** FLIP_S_ mRNA expression

We then tested whether the reduction in the FLIP_S_ protein expression caused by TPL originated from regulation of the FLIP_S_ mRNA expression. Our result showed that treatment with TPL at concentrations ranging from 10 to 25 ng/ml enhanced the mRNA level of c-FLIP in Huh7 cells in a dose- and time-dependent manner (Fig. 3), which suggests that treatment with TPL promotes the transcription of c-FLIP.

The transcription of c-FLIP was activated by NF-κB [2] and MAPK p38 was often an upstream trigger of NF-κB activation under stress [17]. We thus examined if p38 was activated by TPL in Huh7 cells. Our result showed that the phosphorylated p38 levels were enhanced after 24 h of treatment with TPL (Supplemental Fig. 2). Then we investigated the effect of activated p38 on the c-FLIP mRNA expression. We found that pretreatment with SB203580, a specific inhibitor of p38, significantly inhibited TPL-induced increase in the c-FLIP mRNA level (Supplemental Fig. 3). These results imply that the transcription of c-FLIP is probably induced through the p38-NF-κB pathway activated by TPL.

### Downregulation of c-FLIP_S_ protein levels induced by TPL was not achieved through proteasome degradation pathway

**Fig. 4:**
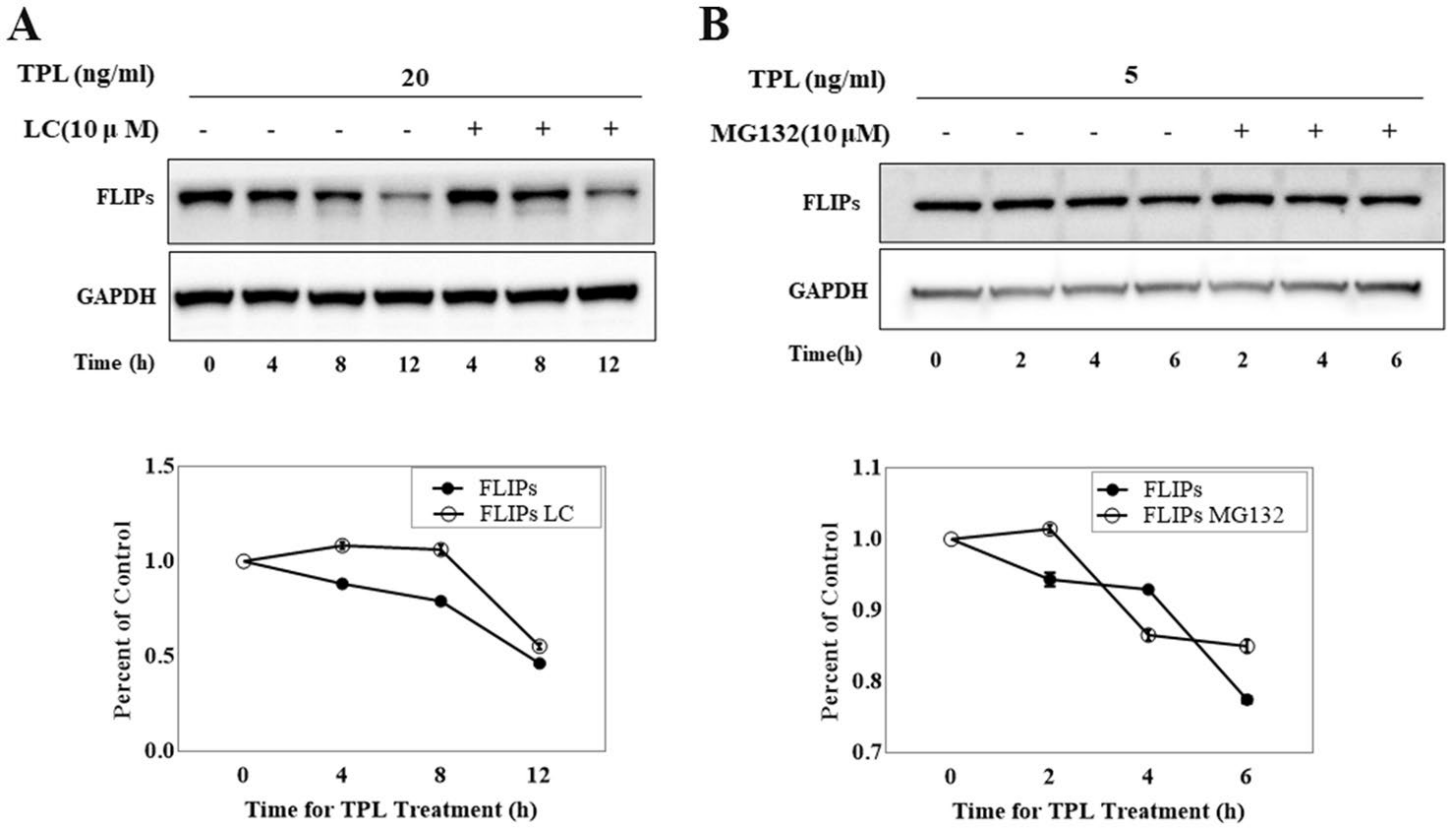
The influence of proteasome inhibitor on FLIP_S_ protein expression. **A** Huh7 cells were treated with 20 ng/ml TPL for 0, 4, 8, 12 h in the absence or presence of 10 μM LC. LC was added 2 h prior to TPL treatment. Total cell lysates were immunoblotted for FLIP_S_ and GAPDH. FLIP_S_ signals were quantitated by densitometric analysis, and the values were expressed as percentage of the control. **B** 10 μM MG132 was added or not in the cell culture 2 h prior to 5 ng/ml TPL which was used to treat cells for 0, 2, 4, 6 h. After that, lysates were prepared for analysis of FLIP_S_ expression

The above data suggest that TPL treatment reduces the protein level of FLIP_S_ by a post-transcriptional mechanism. To investigate whether the TPL-induced decrease in the FLIP_S_ level resulted from proteasome-mediated degradation of FLIP_S_, time-course experiments were carried out for determination of the FLIP_S_ levels after TPL treatment in the absence or presence of the irreversible proteasome inhibitor lactacystein (LC). Figure 4A shows that the protein level of FLIP_S_ was reduced by treatment with TPL time-dependently in the absence of LC (Compare Lane 1 with Lane 2–4). In the presence of LC, the FLIP_S_ protein level was slightly enhanced after 4 h of TPL treatment but reduced significantly after 12 h of treatment (Compare Lane 1 with Lane 5–7). Comparison analysis of the relative protein level of FLIP_S_ between various timepoints and 0 h revealed that FLIP_S_ protein levels were reduced at similar rates by TPL in the absence or presence of LC. Another proteasome inhibitor, MG132, was also used for pretreating Huh7 cells. And we showed that the FLIP_S_ protein level was enhanced after 2 h of treatment with MG132 and 5 ng/ml TPL (Compare Lane 1 and Lane 5 in Fig. 4B) but reduced after 4 or 6 h of treatment (Compare Lane 1 and Lane 6–7 in Fig. 4B). This reduction was similar to the reduction in the FLIP_S_ level in Huh7 cells treated with 5 ng/ml TPL only (Compare Lane 1 and Lane 2–4). This result also demonstrated that the relative level of FLIP_S_ protein was reduced with the increasing time (4, 6 h) for treatment with TPL no matter MG132 was added in the cell culture or not. All these data indicate that, in Huh7 cells, TPL-induced decline in the FLIP_S_ levels is not caused by the proteasome-mediated degradation. Additionally, a caspase-mediated mechanism of FLIP_S_ degradation was ruled out for Fig. 2 displays that the reduction in basal FLIP_S_ level occurred before the caspases were activated.

### Superoxide dismutase (SOD)-mimetic tempol prevented TPL-induced decrease in c-FLIP_S_ levels

Previous study revealed that ROS downregulated the c-FLIP protein expression in cells treated with doxorubicin [18]. To further explore the mechanism by which TPL reduced the c-FLIP_S_ expression, we next investigated the relation of ROS to the TPL-induced downregulation of c-FLIP_S_ protein level. We showed that the ROS levels were significantly increased in Huh7 cells after 24 h of treatment with TPL (Supplemental Table 2 and Supplemental Fig. 4). And Tempol, a SOD mimetic which neutralizes ROS efficiently hindered the decrease in FLIP_S_ protein levels (Supplemental Fig. 5). This result suggests that the increase in the ROS levels produced by TPL treatment accelerates the decrease in FLIP_S_ expression.

## Discussion

Research revealed that apoptosis induced by TNF-α combined with TPL in TNF-α-resistant solid tumor cell lines resulted from the inhibition effect of TPL on the activation of NF-κB induced by TNF-α [1]. But the effect of TPL on basal c-FLIP expression was unknown. In the present study, we found that the combination of TNF-α and TPL promoted apoptosis in Huh7 cells, and we noticed that TPL not only inhibited FLIP_S_ expression induced by TNF-α but also downregulated basal level of FLIP_S_ which was expressed at higher levels in Huh7 cells.

FLIP_L_ and FLIP_S_ are two isoforms of c-FLIP protein. Our western blot analysis results displayed that basal level of FLIP_S_ was much higher than that of the FLIP_L_ in Huh7 cells. This character might be explained by the finding that the expression of c-FLIP isoforms was possibly regulated in a cell line-dependent manner [19–22]. Intriguingly, FLIP_S_ blocks apoptosis exclusively, whereas FLIP_L_ can act as an anti-apoptotic or a pro-apoptotic molecule. FLIP_L_ has a pro-apoptotic role only upon strong receptor stimulation in combination with FLIP_L_ moderate expression [23]. In our study, TPL at lower concentrations increased the expression of FLIP_L_ protein in Hepatoma cells. In addition, the expression of death receptor Fas of Huh7 cells was enhanced after TPL treatment (data not shown). It suggests that the increase in FLIP_L_ protein level induced by TPL may promote apoptosis when the amount of the generated cleavage of products of procaspase-8 is more than that of c-FLIP_L_.

TNF-α upregulated the transcription of c-FLIP by activating NF-κB [2, 24]. We found that, in Huh7 cells, TNF-α increased FLIP_S_ but reduced FLIP_L_ protein expression which might be consistent with the report that JNK activated by TNF-α promoted the proteasomal elimination of FLIP_L_ [25]_._ Lee et al. showed that TPL blocked activation of NF-κB induced by TNF-α [1]. So TPL was supposed to inhibit the FLIP_S_ expression induced by TNF-α when collaborating with TNF-α to induce apoptosis in Huh7 cells. As expected, our result showed that TPL abolished TNF-α-induced increase in FLIP_S_. In addition, basal level of FLIP_S_ was reduced by TPL. At an earlier time, Chen et al. evidenced that TPL sensitized TRAIL resistant pancreatic cancer cells by inducing the downregulation of c-FLIP [16]. This finding supported our result above.

Subsequently, we revealed that decrease in FLIP_S_ brought out by TPL was not achieved by transcriptional regulation. We showed that c-FLIP mRNA level was not reduced but increased by TPL treatment, which indicated that basal NF-κB activity was activated by TPL. We next further confirmed TPL-mediated enhancement in basal NF-κB activity by demonstrating the activation of upstream MAPK p38 induced by TPL. Our finding was supported by Lee et al. They showed that TPL slightly induced NF-κB-mediated transcription in MCF-7 cells [1].

Interestingly, our results also suggest that downregulation of FLIP_S_ caused by TPL does not rely on proteasome-mediated degradation. We showed that proteasome inhibitors failed to prevent TPL-induced decrease in FLIP_S_. This phenomenon was like that found in a study, which showed that FLIP_S_ reduction caused by doxorubicin in prostate cancer cells appeared at the post-transcriptional level independently of proteasome-mediated pathway [15]. Then, we revealed that, in Huh7 cells, overproduction of ROS induced by TPL was involved in downregulation of FLIP_S_. But the mechanism by which ROS reduced FLIP_S_ levels is to be elucidated.

In short, we demonstrated that TNF-α combined with TPL promoted apoptosis in Huh7 cells and TPL not only inhibited the expression of FLIP_S_ induced by TNF-α but also induced downregulation of basal level of FLIP_S_. Furthermore, we showed that TPL reduced basal level of FLIP_S_ through neither suppressing transcription nor inducing degradation. This finding suggested another possible mechanism by which TPL increased sensitivity of tumor cells to TNF-α-induced apoptosis.

## Supplementary Information

Below is the link to the electronic supplementary material.

Supplementary file1 Supplemental Fig. 1 Apoptosis induced by the combination of TNF-α and TPL. Huh7 cells were treated with 5 ng/ml TPL or/and 5 ng/ml TNF-α for about 43 hr. Cell lysates were prepared for Western blot analysis of active caspase-3 and caspase-8, as well as cleaved PARP. GAPDH was used as a loading control. Identical loading controls were used in the subsequent western blot experiments unless otherwise stated. Control: Con (TIF 731 KB)

Supplementary file2 Supplemental Fig. 2 Effect of TPL on the protein levels of phosphorylated p38 (p-p38). Huh7 cells were exposed to 20 ng/ml TPL for various time intervals (0, 6, 12, 24 h). P-p38 levels were assayed by immunoblotting. This data is representative of at least two reproducible experiments (TIF 571 KB)

Supplementary file3 Supplemental Fig. 3 Effect of p38 inhibitor SB203580 on c-FLIP mRNA expression. Huh7 cells were treated with 20 ng/ml TPL for 24 h or 30 h in the presence or absence of 10 μM SB203580. SB203580 was added 2 h prior to TPL treatment. Total RNA was reverse transcribed and then c-FLIP mRNA expression was quantitated by real-time PCR. GAPDH was included as an internal reference. Relative expression of c-FLIP mRNA was expressed as mean ± S.D. from three independent experiments, ** P < 0.01, compared with the treatment with TPL only. (A) FLIPL mRNA expression; (B) FLIPs mRNA expression (TIF 1826 KB)

Supplementary file4 Supplemental Fig. 4 Elevation of ROS induced by TPL treatment. Huh7 cells were treated with TPL (20 ng/ml) for 24 h and then incubated with medium containing H2-DCFDA (10 μM) for 1 h. Then, cells were analyzed with flow cytometry for ROS levels estimate (TIF 762 KB)

Supplementary file5 Supplemental Fig. 5 The effect of Tempol on FLIPs protein expression. (A) Huh7 cells were treated with TPL (20 ng/ml) in the absence or presence of Tempol (1 mM) for 8 h. Cell lysates were immunoblotted with mAb against c-FLIP. Then the FLIPs blot was stripped and immunoblotted for GAPDH. The data is representative of at least two reproducible tests. (B) Comparison analysis of FLIPs densitometry value among Huh7 cells untreated or treated with Tempol, TPL, Tempol combined with TPL. ** P < 0.01, compared with TPL treatment (TIF 1754 KB)

Supplementary file6 (DOCX 15 KB)

## Data Availability

The datasets generated during the current study are not publicly available due to the study has not been published but are available from the corresponding author on reasonable request.
