# Supplementary figures and images for: Triptolide-mediated downregulation of FLIPS in hepatoma cells occurs at the post-transcriptional level independently of proteasome-mediated pathways
Source: Med Oncol. 2022 Oct 29;40(1):7. doi: 10.1007/s12032-022-01857-y (PMC9617966; doi:10.1007/s12032-022-01857-y)

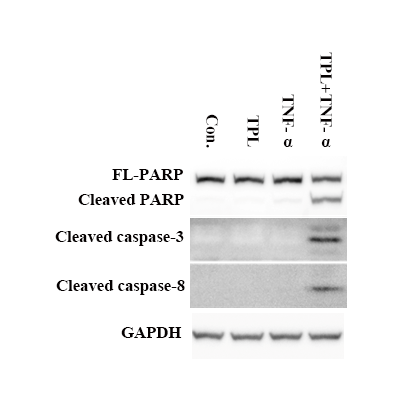

Supplement: Supplementary file 1 — Supplementary file1 Supplemental Fig. 1 Apoptosis induced by the combination of TNF-α and TPL. Huh7 cells were treated with 5 ng/ml TPL or/and 5 ng/ml TNF-α for about 43 hr. Cell lysates were prepared for Western blot analysis of active caspase-3 and caspase-8, as well as cleaved PARP. GAPDH was used as a loading control. Identical loading controls were used in the subsequent western blot experiments unless otherwise stated. Control: Con (TIF 731 KB) [file 12032_2022_1857_MOESM1_ESM.tif]

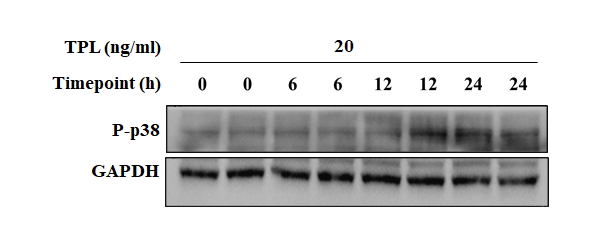

Supplement: Supplementary file 2 — Supplementary file2 Supplemental Fig. 2 Effect of TPL on the protein levels of phosphorylated p38 (p-p38). Huh7 cells were exposed to 20 ng/ml TPL for various time intervals (0, 6, 12, 24 h). P-p38 levels were assayed by immunoblotting. This data is representative of at least two reproducible experiments (TIF 571 KB) [file 12032_2022_1857_MOESM2_ESM.tif]

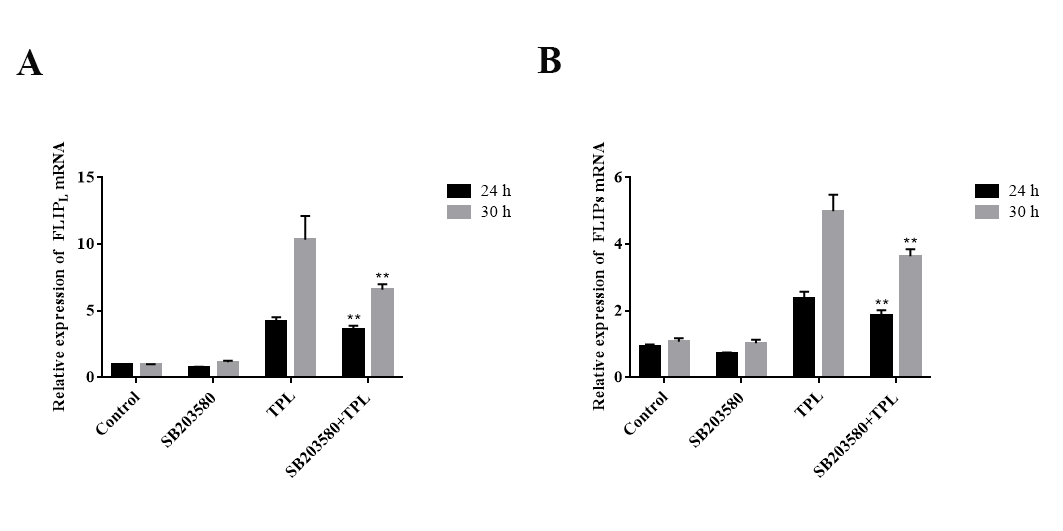

Supplement: Supplementary file 3 — Supplementary file3 Supplemental Fig. 3 Effect of p38 inhibitor SB203580 on c-FLIP mRNA expression. Huh7 cells were treated with 20 ng/ml TPL for 24 h or 30 h in the presence or absence of 10 μM SB203580. SB203580 was added 2 h prior to TPL treatment. Total RNA was reverse transcribed and then c-FLIP mRNA expression was quantitated by real-time PCR. GAPDH was included as an internal reference. Relative expression of c-FLIP mRNA was expressed as mean ± S.D. from three independent experiments, ** P < 0.01, compared with the treatment with TPL only. (A) FLIPL mRNA expression; (B) FLIPs mRNA expression (TIF 1826 KB) [file 12032_2022_1857_MOESM3_ESM.tif]

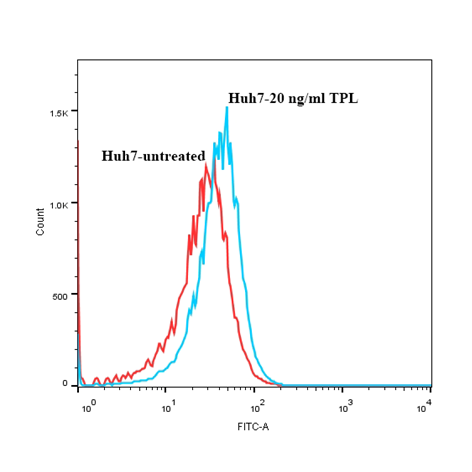

Supplement: Supplementary file 4 — Supplementary file4 Supplemental Fig. 4 Elevation of ROS induced by TPL treatment. Huh7 cells were treated with TPL (20 ng/ml) for 24 h and then incubated with medium containing H2-DCFDA (10 μM) for 1 h. Then, cells were analyzed with flow cytometry for ROS levels estimate (TIF 762 KB) [file 12032_2022_1857_MOESM4_ESM.tif]

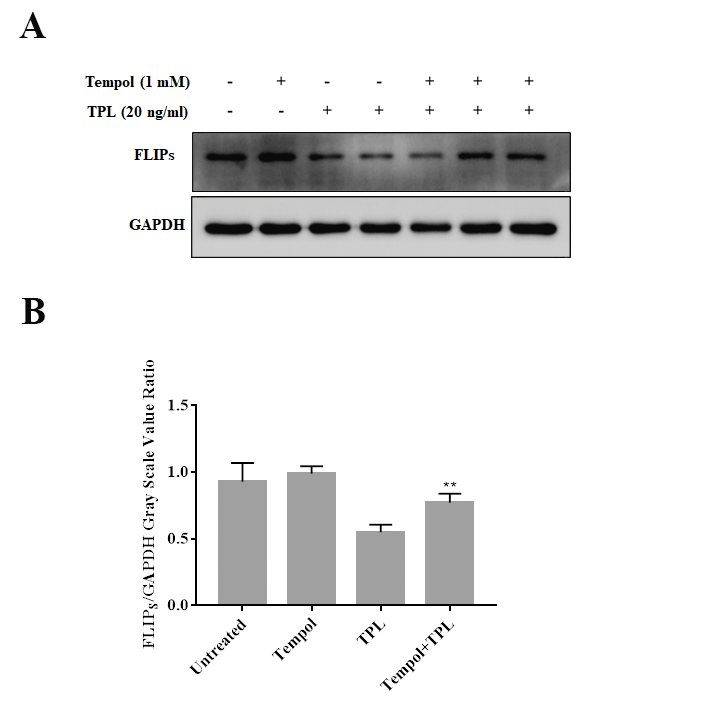

Supplement: Supplementary file 5 — Supplementary file5 Supplemental Fig. 5 The effect of Tempol on FLIPs protein expression. (A) Huh7 cells were treated with TPL (20 ng/ml) in the absence or presence of Tempol (1 mM) for 8 h. Cell lysates were immunoblotted with mAb against c-FLIP. Then the FLIPs blot was stripped and immunoblotted for GAPDH. The data is representative of at least two reproducible tests. (B) Comparison analysis of FLIPs densitometry value among Huh7 cells untreated or treated with Tempol, TPL, Tempol combined with TPL. ** P < 0.01, compared with TPL treatment (TIF 1754 KB) [file 12032_2022_1857_MOESM5_ESM.tif]
