# Supplementary material for: Triptolide-mediated downregulation of FLIPS in hepatoma cells occurs at the post-transcriptional level independently of proteasome-mediated pathways
Source: Med Oncol. 2022 Oct 29;40(1):7. doi: 10.1007/s12032-022-01857-y (PMC9617966; doi:10.1007/s12032-022-01857-y)
Supplement: Supplementary file 6 — Supplementary file6 (DOCX 15 KB) [file 12032_2022_1857_MOESM6_ESM.docx]

**Supplementary Table 1 Survival of Cells treated with TNF-α or/and TPL**

Huh7 cells were treated with indicated concentrations of TNF-α or/and TPL for 48 hr. Cell survival was determined using CCK-8.

|  | | Cell survival (%) | | |  |
| --- | --- | --- | --- | --- | --- |
| Treatment | Experiment 1 | | Experiment 2 | Experiment 3 | Mean ± S.D. |
| Triptolide 2.5 ng/ml | | 107.50 | 96.69 | 95.18 | 99.79 ± 6.72 |
| Triptolide 5 ng/ml | | 94.50 | 82.59 | 81.22 | 86.10 ± 7.3 |
| Triptolide 20 ng/ml | | 66.77 | 72.56 | 74.70 | 71.34 ± 4.11 |
| TNF-α 5 ng/ml | | 104.86 | 100.39 | 94.99 | 100.08 ± 4.94 |
| TNF-α 10 ng/ml | | 108.95 | 103.92 | 99.45 | 104.10 ± 4.75 |
| TNF-α 10 ng/ml + Triptolide 5 ng/ml | | 68.50 | 68.36 | 55.32 | 64.06 ± 7.57 |

**Supplementary Table 2 Mean Fluorescence Intensity Values representing ROS Levels in Huh7 Cells**

| Group | MFI (FITC-A) | | | |
| --- | --- | --- | --- | --- |
|  | Experiment 1 | Experiment 2 | Experiment 3 | Mean + SD |
| Huh7-untreated | 30.4 | 26.6 | 34.6 | 30.53±4.00 |
| Huh7-20 ng/ml TPL | 44.9 | 39.4 | 40.1 | 41.47±2.99 |
